# Avian Lifespan Network Reveals Shared Mechanisms and New Key Players in Animal Longevity

**DOI:** 10.1111/acel.70156

**Published:** 2025-06-28

**Authors:** Mirko Martini, Giovanni Piccinini, Liliana Milani, Mariangela Iannello

**Affiliations:** ^1^ Department of Biological, Geological and Environmental Sciences University of Bologna Bologna Italy

**Keywords:** aging, birds, comparative genomics, lifespan, molecular evolution

## Abstract

Lifespan is a highly variable life trait across the Tree of Life, governed by complex and multifactorial mechanisms. While some conserved pathways regulating longevity have been identified in various species, the molecular basis of this phenotype is far from being understood. In this context, the adoption of new model species and methods of investigation may offer opportunities to explore the molecular underpinnings of longevity in animals. In this study, we investigated the genomic resources of 141 birds to analyze the molecular evolution underlying extremely long‐ and short lifespans. We show that birds with similar lifespans exhibit convergent evolution in specific genes regardless of body mass and phylogenetic relationship, enabling the construction of a “lifespan network” of protein–protein interactions. This network highlights the interplay between metabolism and cell cycle control as key processes in avian lifespan regulation. This lifespan network not only provides evidence for shared mechanisms of lifespan regulation across different organisms but also enables the identification of new candidates for studying aging, particularly in humans. By integrating multiple evolutionary signals from both extremes of the lifespan distribution, our results show the power of evolutionary and comparative approaches in studying complex traits like longevity, providing new insights into aging research.

## Graphical abstract

In this work, we found that birds with similar lifespans exhibit convergent evolution in specific genes, suggesting that shared evolutionary pressures shape the genetic basis of lifespan similarity across species. By investigating protein–protein interactions across genes evolving convergently in long‐ and short‐lived species, we identified a network that highlights the interplay between metabolism and cell cycle control as key processes in avian lifespan regulation. This lifespan network not only provides evidence for shared mechanisms of lifespan regulation across different organisms but also enables the identification of new candidates for studying aging.

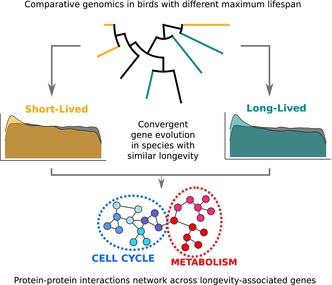

## 1 Introduction

One of the most fascinating unknowns in biology is lifespan regulation. Maximum lifespan (MLS) is a species‐specific trait, with extensions and reductions having evolved independently multiple times in different lineages (Kenyon 2010; Li, Vazquez, et al. 2023). Deciphering the molecular mechanisms underlying this variability is crucial not only for unraveling the complexities of lifespan regulation but also for elucidating aging biology. Aging—the progressive physiological decline with chronological age (Guo et al. 2022)—is inversely correlated with the mean lifespan, as species with extended longevity tend to age more slowly (Li, Vazquez, et al. 2023; Yuan et al. 2023).

In many species, there is a covariation between lifespan and other life‐history traits, such as growth rate, reproductive rate, age at first reproduction, and mortality (Speakman 2005; Miquel et al. 1976; Van Voorhies and Ward 1999; White and Seymour 2004; Promislow and Harvey 1990; Speakman et al. 2002). According to the disposable soma theory (Kirkwood 1977), organisms have limited energetic resources, so there is a trade‐off between resources allocated to reproduction and somatic maintenance. Following this interpretation, species showing high survival rate are believed to invest more energy in self‐maintenance, delaying aging and achieving a longer lifespan at the expense of growth and reproductive rate. Differently, species showing a low survival rate are believed to invest more resources in rapid growth and reproduction. Such faster pace‐of‐life would then lead to a faster accumulation of cytotoxic molecules and cellular damages, resulting in accelerated aging and shorter lifespan.

Investigating genes and molecular pathways influencing lifespan in long‐ and short‐lived species can pave the way for novel strategies to modulate aging. So far, most studies in the aging field have focused on model species with noticeably short lifespans, such as yeasts, fruit flies, nematodes, and mice (Kenyon 2010). These efforts have uncovered numerous shared aging‐related genes and pathways that influence lifespan across species, such as the insulin/insulin‐like growth factor 1 (IGF‐1) signaling pathway and the mechanistic target of rapamycin (mTOR) pathways (Kenyon 2010). While these works have pioneered aging research, how findings in short‐lived model organisms can be transferred to long‐lived species, such as humans, remains unclear (Bertile et al. 2023; Valenzano et al. 2017).

Thanks to advances in high‐throughput sequencing techniques, the growing availability of genomic and transcriptomic data has enabled the study of non‐canonical species, including those with remarkable longevity (Stenvinkel and Shiels 2019). Comparative genomic approaches in mammals (Farré et al. 2021; Keane et al. 2015; Li, Vazquez, et al. 2023; Sahm et al. 2018; Tejada‐Martinez et al. 2022), rockfishes (Treaster et al. 2023), and bivalve mollusks (Iannello et al. 2023) have revealed shared evolutionary signals, such as convergent evolution, positive selection, or duplication, in genes associated with extended lifespans. These studies have identified genes involved in processes like efficient prevention and handling of error accumulation acting at DNA, RNA, and protein levels, offering insights into the common mechanisms that may underlie longevity by slowing aging (Tian et al. 2017). However, our comprehension of lifespan regulation and aging biology remains far from complete, mainly because of process complexity (Shadyab and LaCroix 2015). Moreover, distinguishing species‐specific adaptations from conserved mechanisms adds one level of complexity in cross‐species comparisons (Valenzano et al. 2017). As a result, an exhaustive characterization of molecular players involved in lifespan regulation and aging is lacking, and new approaches and investigational units are required to unravel such phenotypes.

In such a context, birds have emerged as intriguing study subjects. The Aves class shows an extraordinary range of MLSs: from 3 years for the red‐faced warbler (*Cardelilina rubrifrons*—Passeriformes) to 84 years for the American flamingo ( *Phoenicopterus ruber* —Phoenicopteriformes). Moreover, despite showing specific physiological features that are expected to accelerate aging (including particularly high metabolic rates and body temperature, and lower insulin sensitivity) (Travin and Feniouk 2016), birds generally live two to three times longer than similarly sized mammals (Austad 2011), making them an exciting case study to investigate the mechanisms of lifespan variations and aging. In this study, we investigated 141 bird genomes to identify genes underlying lifespan regulation. Specifically, we tested the hypothesis that species with similar lifespans exhibit convergent evolution in certain genes. By integrating molecular evolution analysis with protein–protein interaction inference, we obtained a comprehensive network that enables us to investigate the complex interplay of genes and pathways associated with lifespan in avian species. We found that this network is strongly enriched for factors with known roles in the lifespan of model species, including humans, suggesting that the methods used in this work are effective in identifying players in longevity, opening the possibility to transfer our findings to other species. Notably, we hypothesize that hub genes identified in this network and not yet investigated for aging processes represent new excellent candidates for studying lifespan regulation and aging, also in humans.

## 2 Results

### 2.1 Longevity Records and Genomic Resources

To investigate convergent evolution associated with longevity traits, we collected available maximum lifespan (MLS) records, weights, and annotated genomes for avian species. More in detail, we retrieved MLS and weight records for 1189 avian species (see Data S1 and S2). The median MLS of this dataset is 15 years (interquartile range, IQR: 10–24.5 years), while the median weight is 130 g (IQR: 27.9–637 g). The equation MLS = 5.5027 × weight^0.2082^, with *R* ^2^ = 0.4456 (Figure S1 and Table S1), describes the exponential positive correlation between MLS and weight, and it remains significant even after correcting for phylogenetic relatedness by applying the phylogenetic independent contrast (PIC) method.

**FIGURE 1:**
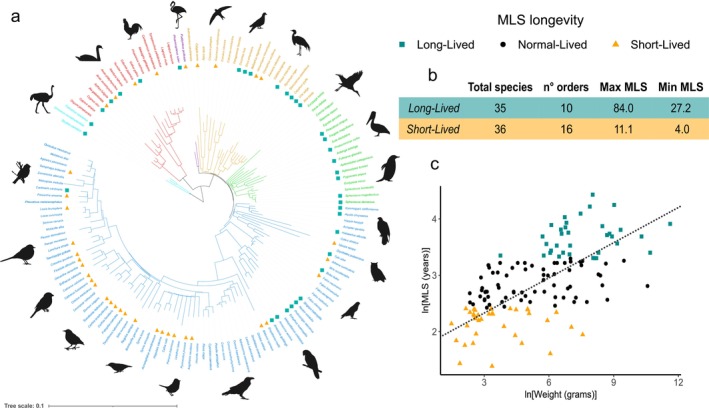
Avian species tree and lifespan distribution characterization. (a) Phylogenetic tree of the studied species based on the topology from Kuhl et al. (2021). The branch lengths are proportional to the average substitution rates in bird protein sequences. Colors are used to identify macro groups of birds, even if not at the same taxonomic hierarchy level. The colors represent cyan for Palaeognathae, red for Galloanserae, purple for Mirandornithes, yellow for Litusilvanae and Columbaves, green for Phaethoquornithes, and blue for Telluaves (which includes the order Passeriformes); (b) A summary table describes statistics for long‐ and short‐lived species. The columns, in order, display the total number of species categorized as long‐ or short‐lived, the number of taxonomic orders these species belong to, and the maximum and minimum MLS values; (c) A log–log plot illustrates the mathematical covariation between MLS and weight values. The dotted line represents the linear regression model automatically fitted by ggplot. Teal squares indicate long‐lived species; orange triangles mark short‐lived species.

From the 1189 species with MLS and weight records, we selected 141 species (Figure 1) based on the criterion reported in Methods (for the complete list and genome accession numbers, see Data S3; median of BUSCO completeness: 86.4%; Data S4) (Manni et al. 2021).

Among them, 35 were classified as long‐lived (MLS > 27) and 36 as short‐lived (MLS ≤ 11) based on the upper and the lower 25^th^ percentiles, respectively, of the MLS distribution in our dataset (Data S5). Long‐lived species were distributed across 16 diverse orders, with notable concentrations in Sphenisciformes (penguins and related species) and Psittaciformes (parrots and related species). In contrast, short‐lived species were found in 10 orders, with two‐thirds belonging to Passeriformes.

### 2.2 Convergent Evolution in Genes Is Associated With Extension and Reduction of Lifespan in Birds

**FIGURE 2:**
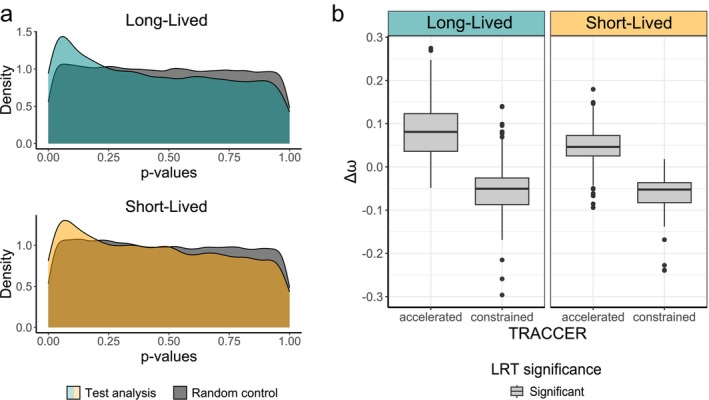
Graphical representation of TRACCER and codeml results. (a) Teal and orange density curves represent the distribution of the *p*‐values for “test analyses” (i.e., conducted on long‐ or short‐lived species). In contrast, the gray curve corresponds to the cumulative distribution of 10 random controls per each test analysis. (b) Box plots show the distribution of the difference between the ω value for trait‐bearing species branches and nontrait‐bearing species branches when the model T2—with three allowed ω classes—is statistically more fitting than the T1 model—with two ω classes allowed—(See Methods). Δω distributions are shown separately for the longevity trait (long‐ vs. short‐lived) and the evolutionary trajectory computed by TRACCER (“constrained” vs. “accelerated”).

A total of 12,322 single‐copy orthologues (OGs) in amino‐acid format were tested for convergent evolutionary rates with TRACCER (Treaster et al. 2021). When tagging trait‐bearing species (long‐ and short‐lived birds, from now on “test analyses”), we observed an enrichment for significant low *p*‐values (*p*‐value < 0.05) in both these analyses when compared to random controls (i.e., analyses on groups of equal size formed by randomly chosen species) (Figure 2 and Data S6–S9). This enrichment in significant *p*‐values means that a higher‐than‐expected number of genes show convergent evolutionary signals in the multiple occurrences of extended or reduced lifespans across our species, a signal that regarded both lower and higher evolutionary rates (respectively “constrained” and “accelerated” genes).

After correction for systematic biases, such as phylogeny and body size (weight is positively correlated with lifespan, see Methods and Data S10–S13), we ended up with 923 convergently evolving genes in long‐lived species (608 “constrained”, 315 “accelerated”), and 511 in short‐lived species (210 “constrained”, 301 “accelerated”).

To evaluate whether the observed constraint or acceleration of evolutionary rates corresponded to specific natural selection forces—purifying selection, and positive or relaxed selection, respectively—we compared codeml models with dN/dS (ω) values constrained into two or three classes (see Methods and Data S14). Through this analysis, we identified 424 genes with concordant TRACCER and codeml results in long‐lived species: 222 genes exhibited lower ω values, indicating stronger purifying selection in trait‐associated species compared to others, while 202 genes showed higher ω values, suggesting positive or relaxed selection in these species. Similarly, we found 249 genes with concordant results in short‐lived species: 138 genes under stronger purifying selection and 111 under positive or relaxed selection in trait‐associated species (Figure 2). Finally, the two sets of concordant genes inferred in long‐ and short‐lived species shared 38 elements. Notably, these shared genes exhibited opposite patterns of selective pressure depending on the longevity phenotype—when under stronger purifying selection in one group, they were under positive or relaxed selection in the other.

Functional enrichments (Gene Ontology—GO—terms) in the two sets of genes associated with long‐lived and short‐lived species are reported in Data S15 and S16. Although the overlap between genes associated with long‐ and short‐livedness is not significant, some functions are similarly enriched in the two groups of convergent genes. These shared functions suggest that, while different gene evolutions are associated with opposite phenotypes, such genes belong to similar pathways. For this reason, we merged the two groups of genes resulting from opposite longevity phenotype analyses (*N* = 635), and we inferred and explored the network of their functional and physical protein–protein interactions, trying to understand their interplay.

### 2.3 The Lifespan Network

By constructing protein–protein interaction (PPI) networks with STRING (Szklarczyk et al. 2015), we found an enrichment in the number of connections in our dataset (number of nodes in networks: 209; number of edges: 207; number of expected edges: 184; enrichment *p*‐value of 0.0487). Specifically, 32.7% of our genes were comprehended in a total of 40 PPI subnetworks, of which the majority have a low number of connections (median number of connections in subnetworks = 2).

**FIGURE 3:**
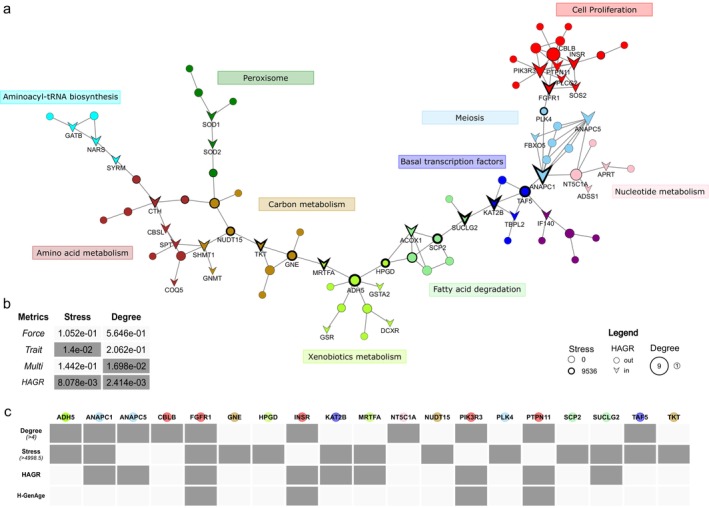
Avian lifespan network. (a) Network of protein–protein interactions across lifespan‐associated genes in birds. Nodes represent proteins, edges represent interactions. Colors correspond to the 11 modules inferred using the k‐mean clustering algorithm in STRING. Each module is labeled with a description of its most common and general function, based on GO and KO terms enrichments. The purple module is an exception, as no clear functional grouping could be identified for the genes within it. To improve network readability, only the protein names of genes included in the HAGR databases or those considered of interest based on their Stress and Degree (see Tables 1 and 2) are displayed. Arrow‐shaped nodes represent proteins included in the HAGR databases, while circular nodes represent proteins not included in it. The size of the node and thickness of the frame encode Degree and Stress value, respectively; (b) The table displays *p*‐value from the Kolmogorov–Smirnov test, which was used to assess the contribution from different gene categories to the network structure. Dark gray indicates significant *p*‐values (*p*‐value < 0.05). The categories evaluated for significantly different contribution in Stress and Degree include genes: associated to long‐ vs. short‐lived species (Trait); characterized by accelerated vs. constrained evolution (Force); shared in both longevity phenotypes vs. specific to only one longevity phenotype (Multi), inclusion vs. exclusion in the HAGR databases; c) The table summarizes information for proteins identified as of interest based on their Stress and Degree. Dark gray tiles identify whether a protein belongs to one or more of the following groups: Degree above the 85^th^ percentile (≥ 4), Stress above the 85^th^ percentile (> 4998.5), included in the HAGR databases, included in the H‐GenAge database.

We focused our analysis on the most extensive one, comprising 86 genes, which we will refer to as the “lifespan network” (Figure 3, Figure S2 and Data S17). Within it, we identified genes showing the highest values of “Degree” and “Stress,” which are particularly informative of core genes of the network (see Methods). Genes with a high Degree represent those with the greatest number of edges connecting them to their direct neighbors; therefore, they are an indicator of core nodes in the modules. Genes with a Degree above the *85^th^* percentile of the distribution (Degree ≥ 5) are listed in Table 1. On the other hand, the value of Stress is directly proportional to how much a node is necessary to maintain connections among nodes; in other words, it is an indicator of nodes with key “bridge” roles in the networks. Genes with a Stress value above the *85* ^*th*^ percentile (Stress > 4998.5) are listed in Table 2.

**TABLE 1:** Genes in the lifespan network with Degree above the 85^th^ percentile of the distribution. Genes with Degree higher than or equal to 5 are listed in descending order. The columns display the protein symbol of the gene product, the complete protein name, the number of connections with neighboring nodes (Degree), the selective force inferred, and the longevity trait of the group where the selective force acts. Proteins marked with an asterisk (*) are also significant based on Stress distribution.

Protein symbol	Description	Degree	Force	Trait
ANAPC1*	Anaphase promoting Complex subunit 1	9	Constrained	LL
PIK3R3	Phosphoinositide‐3‐kinase regulatory subunit 3	8	Constrained	SL
ANAPC5	Anaphase promoting complex subunit 5	7	Constrained	LL
CBLB	Cbl proto‐oncogene B	7	Constrained	LL
INSR	Insulin receptor	6	Constrained	LL
ADH5*	S‐(hydroxymethyl)glutathione dehydrogenase/alcohol dehydrogenase	6	Constrained	SL
TAF5*	Transcription initiation factor TFIID subunit 5	5	Accelerated	LL
NT5C1A	5′‐nucleotidase, cytosolic IA	5	Accelerated	SL
FGFR1*	Fibroblast growth factor receptor 1	5	Constrained	SL
PTPN11	Tyrosine‐protein phosphatase non‐receptor type 11	5	Constrained	LL

Abbreviations: LL = long‐lived species; SL = short‐lived species.

**TABLE 2:** Genes in the lifespan network with Stress above the 85^th^ percentile of the distribution. Genes with Stress higher than 4998.5 are listed in descending order. The columns display the protein symbol of the gene product, the complete protein name, the Stress value, the selective force inferred, and the longevity trait of the group where the selective force acts. Proteins marked with an asterisk (*) are also significant based on Degree distribution.

Protein symbol	Description	Stress	Force	Trait
ADH5*	S‐(hydroxymethyl)glutathione dehydrogenase/alcohol dehydrogenase	9,536	Constrained	SL
SCP2	Sterol carrier protein 2	9,352	Constrained	SL
SUCLG2	Succinate‐CoA ligase GDP‐forming subunit beta	9,198	Accelerated	SL
HPGD	15‐hydroxyprostaglandin dehydrogenase	9,184	Constrained	SL
KAT2B	Lysine acetyltransferase 2B	8,932	Accelerated	LL
TAF5*	Transcription initiation factor TFIID subunit 5	8,930	Accelerated	LL
ANAPC1*	Anaphase promoting complex subunit 1	8,492	Constrained	LL
MRTFA	Myocardin related transcription factor A	7,744	Accelerated	SL
GNE	Bifunctional UDP‐*N*‐acetylglucosamine 2‐epimerase/*N*‐acetylmannosamine kinase	7,624	Accelerated	SL
TKT	Transketolase	6,804	Accelerated	LL
NUDT15	Putative 8‐oxo‐dGTP diphosphatase NUDT15	6,704	Constrained	SL
PLK4	Polo like kinase 4	5,980	Accelerated	SL
FGFR1*	Fibroblast growth factor receptor 1	5,822	Constrained	SL

Abbreviations: LL = long‐lived species; SL = short‐lived species.

We then analyzed network metrics to assess the contributions of genes belonging to different categories to the network structure (see Methods for the investigated categories). Although we generally found no significant differences in Stress and Degree among gene sets under most gene categories (Figure 3) or their combinations (Data S18), we observed notable exceptions. First, genes identified as convergently evolving in short‐lived species exhibited significantly higher Stress levels than those from long‐lived species (Kolmogorov–Smirnov test, *p*‐value: 0.01400; Figure 3 and Data S19). Second, genes present in HAGR, a curated database collection of genes with roles in aging and longevity regulation, showed significantly higher Degree and Stress levels than those not included in this database (Kolmogorov–Smirnov test, *p*‐values: 0.002413 and 0.008078, respectively; Figure 3 and Data S19). Notably, genes in the HAGR databases are significantly enriched in our network compared to the entire lifespan genes dataset and the complete orthogroups dataset analyzed in this study (χ^2^ test, *p*‐values < 0.00001; Tables S2 and S3 and Data S20 and S21). It is worth highlighting that the interactions are biased toward well‐studied genes, particularly those associated with aging and aging‐related diseases (Fernandes et al. 2016). As such, it is possible that the contribution in our network of genes with a known role in longevity is overestimated, while potentially important but underexplored genes could be overlooked due to a lack of experimental evidence for interactions.

### 2.4 Metabolism and Cell Cycle Control Are Major Components in the Lifespan Network

With the k‐means clustering algorithm implemented in STRING, we identified 11 modules constituting the lifespan network (Figure 3 and Data S22).

The distribution of modules and their principal enriched pathways reveal two major components. On the left side of the network, modules cyan, green, brown, sienna, green‐yellow, and light green are associated with metabolic pathways and are enriched for lipid beta‐oxidation, xenobiotic degradation, and t‐RNA metabolism (Figure 3). On the right side of the network, modules blue, purple, pink, light blue, and red are instead enriched for genes involved in cell cycle and cell proliferation control (Figure 3). A detailed list of all enriched pathways and GO terms, including associated *p*‐values and module identifiers, is provided in Data S23 and S24.

GO enrichment analyses of the complete network (Data S24) corroborate these findings and highlight significant biological processes associated with both metabolic processes (“fatty acid beta‐oxidation using acyl‐CoA oxidase”, “cellular lipid metabolic process”, and “phosphate‐containing compound metabolic process”) and cell proliferation and cycle control (“tRNA aminoacylation for protein translation”, and “anaphase‐promoting complex‐dependent catabolic process”).

## 3 Discussion

The incredible gap in knowledge about lifespan regulation demands the adoption of new strategies and methods to identify genes involved in this mechanism and their complex interplay. One promising solution is comparative evolutionary biology, which allows us to take advantage of the astonishing lifespan variability present in nature and seek genomic signatures that may explain it.

In this work, we used a multi‐strategy pipeline (including investigation of convergent evolution, codon‐based selection analyses, and inference of protein–protein interactions network) to identify genes and pathways acting on bird lifespan regulation. We focused on identifying genes that evolved convergently in long‐ and short‐lived birds, testing the hypothesis that convergent evolution of such genes may underlie longevity phenotypes. We then tested if this pipeline could confirm already known players in the aging process and identify new candidate genes that regulate the lifespan of various organisms, including humans.

By analyzing protein evolution in birds with different lifespans, we confirmed our hypothesis that convergent short‐ or long‐livedness in birds is associated with the convergent evolution of a set of genes. Such evidence supports the hypothesis that shared evolutionary pressures shape the genetic basis of lifespan similarity across species. To limit the incidence of false positives in our results—a common downside of evolutionary methods—we adopted different countermeasures (see Methods) and we focused our attention on genes showing both convergent protein evolution and convergent selective pressure in species sharing a similar longevity phenotype. Considering the similarly enriched functions in genes associated with opposite longevity phenotypes, which suggests a co‐participation of such genes in similar pathways, we inferred and explored the network of their functional and physical protein–protein interactions, trying to understand their interplay. This network resulted significantly enriched in interactions, supporting the fact that these genes are involved in the same biological processes, possibly involved in lifespan regulation in birds.

Our network inference reveals that both genes inferred from the long‐lived analysis and genes identified in the short‐lived analysis contribute to the network structure. So far, comparative evolutionary works focused on long‐lived species only, thus identifying genetic signatures limited to lifespan extension (Farré et al. 2021; Iannello et al. 2023; Keane et al. 2015; Li, Wang, et al. 2023; Treaster et al. 2023). Here, we show that integrating signals from long‐ and short‐lived species further contributes to unraveling the molecular players underlying lifespan regulation. As a matter of fact, genes identified in short‐lived species, having higher Stress values, appear to have a crucial role in connecting different modules of the network. Therefore, the two extremes of the longevity spectrum are complementary in exploring longevity using evolutionary approaches. Similarly, in the analysis of genes with a role in lifespan and longevity, past works have mainly focused on genes showing positive selection (Keane et al. 2015; Sahm et al. 2018; Tejada‐Martinez et al. 2022), and the contribution of constrained evolution in shaping such a process has been mostly overlooked. Here, we highlight that both evolutionary forces may contribute to unraveling lifespan regulation mechanisms.

Notably, the lifespan network we identified is strongly enriched for genes that were previously associated to lifespan regulation in humans and model species, i.e., HAGR genes, supporting the hypothesis that some lifespan regulation mechanisms are shared and conserved across different taxa (Curran and Ruvkun 2007; Tian et al. 2017). Our results have two implications. First, they support the idea that our pipeline successfully identifies genes associated with lifespan regulation, removing false positives. Second, they highlight the transferability of our findings to other species, enabling the identification of new common players in lifespan regulation and aging.

By exploring the network of protein–protein interactions, two main functional components emerge. These include cell cycle control and the metabolism of multiple compounds (including nucleotides, amino acids, lipids, carbohydrates, and xenobiotics). Their presence is not surprising since longevity is intimately linked to both, and in general, to the pace‐of‐life (Finkel 2015; Hulbert et al. 2007; Jiménez et al. 2015; Kumari and Jat 2021; López‐Otín et al. 2016; Parkhitko et al. 2020; Postnikoff and Harkness 2012). To date, few works investigated the relationship between life‐history traits and lifespan in birds. Trevelyan et al. (1990) found that there is a correlation between MLS and resting metabolic rate in avian species, suggesting that short‐lived birds generally show higher metabolic rates compared to long‐lived ones. Along with that, some studies have reported that long‐lived bird species tend to have higher antioxidant capacity and experience less oxidative damage than short‐lived species. This suggests that more efficient management or prevention of oxidative stress may contribute to differences in avian lifespans (Vágási et al. 2019; Domínguez‐de‐Barros et al. 2023). In this context, finding genes related to metabolism, cell cycle regulation, and antioxidant capacity that exhibit a specific evolution in either long‐ or short‐lived species may highlight the genetic bases underlying such opposite lifespan phenotypes—and associated life‐history traits—in birds. For example, *Anapc1*, *Anapc5*, and *Fbxo5* (which here show convergent constrained evolution in long‐lived species; Figure S2) are crucial regulators of anaphase onset, either promoting or delaying this delicate phase of mitosis (Schrock et al. 2020; Barford 2011; Verschuren et al. 2007). Given that an unproper segregation of chromosome is associated with genomic instability and aging (Lombard et al. 2005; Karanjawala and Lieber 2004), we hypothesize that constrained selection on these genes in long‐lived species may play a role in avoiding the insurgence of genomic instabilities during cell cycle progression, contributing to lifespan extension. Our analyses additionally support the role of oxidative stress in aging, as we found specific evolution in long‐ and short‐lived species in different genes involved in the metabolisms of harmful radicals. Among these, we highlight the *Gsr* gene, which helps maintaining a high concentration of glutathione in its reduced form (Lüersen et al. 2013), and *Sod1* and *Sod2*, which degrade harmful radicals in mitochondria and cytoplasm. Mutations in these genes are associated with premature aging and cancer (Eleutherio et al. 2021; Carroll et al. 2015; Velarde et al. 2012) and different selection on these genes may reflect the different antioxidant capacity observed between long‐ and short‐lived birds (Vágási et al. 2019; Domínguez‐de‐Barros et al. 2023). Finally, the large metabolic component in our network reflects the intimate link between metabolic rate and lifespan in animals. Among genes identified here, we mention some already associated to aging, such as *Gck*, *Acox1* and *Acox2*, which play key roles in glucose (Abu Aqel et al. 2024) and fatty acid metabolism (Chung et al. 2020; Vamecq et al. 2018; Tan et al. 2024), catalyzing the rate‐limiting step of their respective metabolic pathways.

Overall, although the above‐mentioned genes are interesting individually, it is likely that a combination of multiple, differently selected genes within the network collectively contributes to shaping lifespan toward either short‐ or long‐lived phenotypes. While the precise physiological consequences of different selection on these genes remain difficult to predict based solely on in silico analysis, the interactions identified in the lifespan network may help to elucidate the complex interplay among different genes and pathways co‐involved in the longevity phenotype, highlighting key candidates in this process. In our network, genes showing higher Degree (hub genes) or Stress represent those with a pivotal role in the network, representing the most promising candidates. Among our hub genes, some have a well‐known role in affecting lifespan and aging in different species, and they are enriched for genes with a known role in human aging (Human GenAge—H‐GenAge—, one of the databases part of the HAGR collection). Particularly, *Fgfr1*, *Insr*, *Pik3r3*, and *Ptpn11* play a crucial role in regulating pathways such as mTOR, AMPK, insulin signaling, and IGF‐1, which are known to affect lifespan in distantly related species (Kenyon 2010). Finding such genes as hubs in our lifespan network supports their central role in avian longevity, as well.

The statistical overrepresentation of H‐GenAge genes among our hub genes suggests that other, yet not studied, hub genes in our network are excellent candidates for investigating aging in humans and other species. Among these, we point out two genes showing both high Degree and Stress in the lifespan network: the transcription initiation factor TFIID subunit 5 (*Taf5*) (Bhattacharya et al. 2007)—a component of the basal Transcription factor II D (TFIID), whose function is not yet fully understood—and alcohol dehydrogenase 5 (class III) (*Adh5*) (Reingruber and Pontel 2018)—a member of the alcohol dehydrogenase family, with a key role in the elimination of formaldehyde, a cytotoxic metabolite that induces DNA damage. In addition, other genes in our network show either high Stress or Degree. Among these, we report Cbl proto‐oncogene B (*Cblb*) (Liyasova et al. 2015)—known to interact with proteins involved in immune response and signaling pathways—, Nudix Hydrolase 15 (*Nudt15*) (Carter et al. 2015)—which removes oxidatively damaged nucleosides, preventing their incorporation into DNA—, Polo Like Kinase 4 (*Plk*4LK4) (Bettencourt‐Dias et al. 2005)—which regulates centriole duplication during the cell cycle—, and Transketolase (*Tkt*) (Mitschke et al. 2010)—which play a main role in the pentose phosphate pathway. Although not yet directly investigating aging and longevity, all the above‐mentioned genes show in humans a transcriptional decrease with senescence or following knockout of genes with a role in aging, such as sirtuins, further supporting that their link with aging deserves to be further explored.

Overall, our results support the hypothesis that a shared genetic toolkit underlies lifespan regulation in different animal species. Such a lifespan network provides new attractive candidates that may have a central role in lifespan regulation and aging in multiple species, including humans. Finally, this work highlights the contribution that evolutionary methods may provide in identifying key players of lifespan regulation and aging.

## 4 Methods

### 4.1 Longevity and Weight Records

The dataset of longevity records for birds was sourced from the 14^th^ (de Magalhães et al. 2024) version of the AnAge database downloaded in January 2023 (https://genomics.senescence.info/species/dataset.zip). For each 1189 species, we collected the MLS measures, with no distinction between natural and captive environment values, and “adult weight” (average of adult individuals) (Tacutu et al. 2018). We also added *Anas carolinesis* and *A. zonorhyncha* because they were described as long‐lived in previous research (Harper et al. 2011).

Annotated genomic data used in this project were downloaded in September 2023 from NCBI datasets (Wheeler et al. 2007) and Ensembl standard and Rapid Release databases (Martin et al. 2023). We used scripts from the AGAT toolkit (Dainat and Hereñú 2020) to extract and translate amino acid sequences for the longest isoform of each protein‐coding gene, removing then pseudogenes—intended as sequences with internal stop codons.

The proteome quality was assessed using BUSCO v.5.4.2 with the avian database “aves_odb10”, downloaded in September 2023. We retained only genomes with a completeness percentage equal to or exceeding 65%. We chose this threshold to include *Phoenicopterus ruber*, as it was the longest‐lived species in our dataset (based on MLS).

### 4.2 Longevity Indexes

To distinguish between long‐ or short‐lived birds, we implemented three known longevity measures to determine which was the most effective in detecting any possible convergent signal at the genomic level associated with longevity.

The most intuitive approach for defining longevity was based on MLS distribution, which is the longest recorded lifespan of a species. Birds were categorized as long‐lived (LL_MLS_) if their record lifespan was in the 4^th^ quartile of the MLS distribution (MLS > 27 years) or short‐lived (SL_MLS_) when their record lifespan was in the 1^st^ quartile (MLS ≤ 11.1 years).

Given the positive correlation between MLS and adult weight in vertebrates (Lindstedt and Calder 1976), weight is typically considered a confounding factor when studying longevity. Therefore, we also implemented the “longevity quotient” (LQ) index to account for body mass in the longevity calculation. The LQ index is the ratio between the known MLS and the expected lifespan based on weight. We arbitrarily categorized birds into three groups: long‐lived (LL_LQ_) with values equal to or greater than 2.0 (species living at least twice as long as expected based on their weights), normal‐lived (NL_LQ_) with values between 0.5 and 2.0, and short‐lived (SL_LQ_) with values equal to or less than 0.5 (species living no more than half as long as expected based on their weights).

Finally, following the approach by Kowalczyk et al. (2020), we grouped birds based on a phylogenetic‐aware principal component analysis (p‐PCA) of MLS and weight. In this way, we discriminated between two distinct extended longevity traits: “Long‐Lived Large‐bodied” (3L) and “Exceptionally Long‐Lived given body size” (ELL), defined by the first and second principal components of the p‐PCA, respectively.

### 4.3 Orthology Inference and Branch Length Calculation

Orthology inference was performed using OrthoFinder v2.5.5, with the “‐‐ultra‐sensitive” parameter and the species tree topology from Kuhl et al. (2021).

A total of 10,591 multi‐copy orthogroups (OGs) obtained from OrthoFinder were converted into single‐copy OGs using DISCO v1.3.1 (Willson et al. 2022). Overall, 13,271 single‐copy OGs were retained when they included at least half of the species in the dataset (i.e., 70).

A subset of the single‐copy OGs identified directly by Orthofinder was used to optimize branch lengths of the species tree (Data S24). We extracted only single‐copy OGs that included at least 99% of total species, all LQ_SL_ and LQ_LL_ species, and at least one bird among *Struthio camelus* , *Dromaius novaehollandiae* , and *Casuarius casuarius*, which constituted our internal outgroup (569 OGs in total). First, we aligned each OGs using MAFFT v.7.490 (Katoh and Standley 2013) with the “‐‐auto” option; then, we trimmed the alignments using BMGE v1.12 (Criscuolo and Gribaldo 2010) to enhance the quality of multiple sequence alignments by removing poorly aligned regions through an entropy‐based approach. After the first trimming based on amino acid position conservation (parameters: ‐m BLOSUM30 ‐h 0.5 ‐g 0.4), we removed all sequences with more than 80% of their length constituted by gaps (‐h 1 ‐g 0.8:1). The concatenated alignments of such OGs, constructed with AMAS (Borowiec 2016), was fed to IQTREE v2.2.5 (Minh et al. 2020) for model selection (Kalyaanamoorthy et al. 2017) and partitioning scheme inference (Chernomor et al. 2016) with the parameters “‐m MF + MERGE” and “‐‐rcluster 15”. The obtained partitioning scheme with respective models of evolution was used to infer branch lengths with RAxML‐NG (Kozlov et al. 2019) on the fixed species tree topology of Kuhl et al. (2021).

To perform convergence analyses, branch lengths of gene trees were also required. To do it, we started with single‐copy OGs obtained after DISCO, which were aligned and trimmed as described above. We then extracted only those that included at least 50% of the total species, 50% of LQ_SL_ and LQ_LL_ species, and at least one bird in our internal outgroup. For each single‐copy OG thus filtered (12,322), we inferred branch lengths on the fixed species tree topology of Kuhl et al. (2021), pruning it with the ETE3 toolkit v3.1.3 when necessary (Huerta‐Cepas et al. 2016). Similarly to the previous species tree approach, we inferred the best‐fit model with ModelFinder as implemented by IQTREE (confining the model selection to JTT, Q.bird, and Q.mammal) and optimized branch lengths on the fixed topology with RAxML‐NG.

### 4.4 Convergence Analysis

Convergent rate analyses were performed using TRACCER (Topologically Ranked Analysis of Convergence via Comparative Evolutionary Rates), a tool designed to identify signals of convergent evolution across species with shared traits leveraging branch length comparison of species and gene trees. Specifically, TRACCER calculates Relative Evolutionary Rates (RER) between all pairs of species with and without the trait of interest without inferring any ancestral state. Then, it identifies genes that exhibit significantly different evolutionary patterns in the trait‐bearing species by comparing them with a null expectation based on permutations, labeling genes as either “constrained” or “accelerated.”

After an explorative analysis of the different longevity metrics described in Methods, we decided to proceed using only MLS to label trait‐bearing species. For a detailed description of the reasons and analyses supporting such a decision, see Data S1. We performed two tests, each using species from one of the trait‐bearing groups (long‐lived and short‐lived species labeled using MLS distribution) as the foreground compared against a background composed of the respective nontrait‐bearing species. To test if the possible convergence in our tested species may be due to chance or systematic biases, we compared TRACCER results obtained in short‐ and long‐lived species (test analyses) to three different types of controls. The first control was conceived to test if the number of genes showing convergence in trait‐bearing groups was higher than expected in groups of random species, which are supposed not to share convergent traits. Therefore, we performed a “random control” selecting as many randomly chosen nontrait‐bearing species as trait‐bearing ones. We repeated this random selection 10 times for each test to create a more robust null distribution of the level of convergent signals expected by chance in species sharing no traits. The distributions of TRACCER *p*‐values obtained from these random controls were plotted as a unique and cumulative distribution to account for the possibility of picking convergent traits by chance in randomly chosen species, which could have skewed the significance of our results.

The second control was conceived to consider the possible non‐random phylogenetic distribution of trait‐bearing species. In particular, two‐thirds of the short‐lived species were Passeriformes. Considering such a bias, convergent signals detected in trait‐bearing species may be due to taxon‐specific evolution rather than longevity. For this reason, we performed a “phylogenetic control” by selecting closely related nontrait‐bearing species or, if unavailable, randomly choosing from the closest branches.

The third control was conceived to consider weight as a possible confounding factor in our convergent analyses. Considering that a positive correlation exists between longevity and weight, there is a bias in the weight distribution of short‐ and long‐lived species. For this reason, we performed a “weight control” aimed at identifying genes with potential co‐evolution associated with shared weight characteristics rather than longevity‐related traits. Specifically, species were classified as “heavy” and “light” based on the fourth (adult weight > 1,078 g) and first (adult weight ≤ 25.6 g) quartile of the adult weight distribution, respectively. Finally, we selected heavy species that were not long‐lived and light species that were not short‐lived as foreground groups for two additional convergent analyses.

We removed all genes identified as “significant” in both the phylogenetic and weight control and our test analyses, as we considered them false positives unrelated to the longevity phenotypes. Nonetheless, since we expected several additional false positives among genes identified by TRACCER, we performed a codon‐based selection analysis as a method to reduce their occurrence.

### 4.5 Selection Analysis

To determine if convergent “constrained” or “accelerated” evolution in genes identified with TRACCER was also associated with purifying or accelerated (positive or relaxed) selection, respectively, we calculated dN/dS ratios (ω) using codeml from the PAML suite v.4.10.7 (Yang 2007, 1997). To avoid any assumption on the ancestral state of the trait, we conducted branch‐tests analyses on terminal branches rather than whole clades of trait‐bearing species. For each OGs, we tested the likelihood of two models, one of which was nested into the other: model T1 was allowed to attribute different ωs to terminal vs. inner branches of the tree (two ω categories); model T2 included an additional ω category since terminal branches of trait‐bearing species and those of nontrait‐bearing ones were allowed to have different values. A higher likelihood of T2 vs. T1 (tested with Likelihood Ratio Test, LRT) meant that there is a significant difference between the ωs of the terminal branches of trait‐ vs. nontrait‐bearing species, i.e., they are currently experiencing different selective pressures.

To perform codeml analyses, we extracted nucleotide sequences from downloaded genomes with AGAT and the “‐‐roo*”* flag. We then matched sequence headers to reconstruct nucleotide single‐copy OGs from proteomic single‐copy OGs. Using a custom script, we converted our aligned and trimmed protein OGs into the corresponding aligned and trimmed nucleotide OGs. For genes showing significant results with TRACCER, we then ran, for each OG, codeml. Finally, we conducted a likelihood ratio test to determine which model best described our data, retaining only genes showing significant *p*‐values. We did not apply multiple correction methods, as recommended for a priori selected set of genes by Álvarez‐Carretero et al. (2023). To test the presence of functional enrichments in genes with convergent evolution in long‐ and short‐lived species, we performed a Gene Ontology (GO) enrichment analysis using as background the annotated longest sequence for each single‐copy OG investigated in this work. We used InterProScan v.5.65‐97.0 (Jones et al. 2014) to obtain such GO terms annotation. GO terms enrichment analyses were performed using the “topGO” package in R (Alexa and Rahnenfuhrer 2023) with the “elim” algorithm to eliminate hierarchy‐based redundancy. From now on, the complete set of genes showing both convergent evolution and convergent selection in long‐ and short‐lived species will be called “lifespan genes”.

### 4.6 Network Construction and Analysis

Physical and functional interactions of lifespan genes were analyzed and visualized using STRING (Search Tool for the Retrieval of Interacting Genes/Proteins). We used “databases” and “experiments” as active interaction sources and “high confidence (0.700)” as the minimum required interaction score. We chose *Calidris pygmaea* as the reference species due to the high level of matching sequences.

We focused our subsequent analyses on the largest identified network. Using the k‐means clustering algorithm in STRING, we detected network modules and performed GO term enrichment analysis on the entire network and its modules. Additionally, KEGG orthologous (KO) and KEGG pathways (ko) enrichment analyses were performed on the same genes using the “clusterProfiler” package in R (Wu et al. 2021). The background, defined as before, was annotated with eggNOG (Huerta‐Cepas et al. 2019). We exported the largest STRING network and further analyzed it using Cytoscape 3.10.2 (Shannon et al. 2003). Utilizing the built‐in “Analyze Network” tool, we extracted crucial network metrics commonly used in network characterization (average shortest path length, clustering coefficient, closeness centrality, eccentricity, stress, degree, betweenness centrality, neighborhood connectivity, radiality, topological coefficient). Subsequently, we examined the distribution of these metrics to identify potential patterns. Particularly, we focused our attention on “Degree”—a measure of the number of edges connecting a node to its direct neighbors, therefore an indicator of core nodes in modules—and “Stress”—which quantifies how much a node is necessary to maintain the connection among nodes, therefore an indicator of nodes with key bridge role in the network (Wang et al. 2022). Finally, using the metrics described above, we tested the differential contributions of genes to the network based on (1) different evolutionary forces (accelerated vs. constrained), (2) association with distinct evolutionary traits (long‐lived vs. short‐lived), (3) known roles in longevity regulation in other species (present vs. absent in the HAGR databases), and (4) whether the genes were shared between long‐lived and short‐lived species or specific to only one longevity phenotype.

### 4.7 Cross‐Reference With Senescence and Aging Databases

To test if genes associated with longevity in birds had a role in affecting aging and lifespan in other species, in particular humans, we cross‐referenced our genes of interest with three different databases comprising genes related to aging: CellAge (built 3, downloaded in October 2024), GenAge for humans, and GenAge for model organisms (build 24, downloaded on October 2024), which are part of the HAGR (Human Aging Genomic Resources) database collection. We used KEGG orthogroups (KO) to identify orthology between our genes of interest and aging‐related genes from other organisms. KAAS (KEGG Automatic Annotation Server) (Moriya et al. 2007) was used to annotate all interesting proteins using SBH (Single‐directional Best Hit) as the assigning method after selecting all avian species available in the gene dataset. Conversely, KOs for the HAGR databases were retrieved by converting Entrez gene IDs using the functions “keggConv” and “keggGet” in the “KEGGREST” package in R (Tenenbaum and Maintainer 2023). When no KO term was associated with our genes of interest, DIAMOND v.2.0.6 (Buchfink et al. 2021) with the “‐‐ultra‐sensitive” mode against NCBI's non‐redundant protein database (nr) was used to complete the gene annotations. NCBI sequence identifiers were then used to automatically designate protein symbols and names using the E‐utilities tool from Entrez Direct provided by NCBI (Kans 2024).

Finally, we manually curated all these lists of proteins to ensure their completeness. Specifically, to discard paralogs, we retained only those proteins that matched their KO IDs and names. This last part was not performed with the database GenAge for model organisms.

### 4.8 Statistical Analyses

We conducted PIC on MLS and weight values to investigate whether an evolutionary link exists between these two metrics beyond their mathematical correlation. This analysis was performed using the function “PIC” in “ape” package in R (Paradis and Schliep 2019).

To select the best‐fitting model for our data in codeml analyses, we applied a likelihood ratio test (LRT) performed using the function “chi2” from the “scipy.maths” library in Python (Virtanen et al. 2020).

For enrichment significance analysis of HAGR genes against various backgrounds, we performed a χ^2^ test with the functions “TEST.CHI” and “INV.CHI” in Microsoft Excel for Microsoft 365 MSO (Version 2411 Build 16.0.18227.20082) 64 bit.

Finally, we evaluated the significance of differences in network metrics distributions using the function “wilcoxon.test” in “stats” package of R (R Core Team 2024).

## Author Contributions

M.M. and M.I. designed the study. M.M. and M.I. performed formal analyses. M.M., M.I., and G.P. investigated the data. M.M., G.P., L.M., and M.I. discussed the results. M.M. curated the data and the repository online. M.M. visualized the data. L.M. provided computational resources and fundings. M.M. and M.I. wrote the first version of the manuscript. M.M., G.P., L.M., and M.I. revised the manuscript. M.M., G.P., L.M., and M.I. read and approved the final version of the manuscript.

## Conflicts of Interest

The authors declare no conflicts of interest.

## Supporting information


**Data S1.** Results of the χ^2^ test for the overrepresentation of genes included in the HAGR databases among genes of interest. We tested the overrepresentation of genes included in the HAGR database present in our lifespan network compared to those in all.

**Figure S1.** Covariation between maximum lifespan and weight values. A log–log plot illustrates the mathematical.
**Figure S2.** Avian lifespan network. Network of protein–protein interactions across lifespan‐associated genes.

## Data Availability

All data and codes required to reproduce this work are available in the GitHub repository https://github.com/MirkMart/Avian‐Lifespan‐Network.git.
