# Supplementary material for: Avian Lifespan Network Reveals Shared Mechanisms and New Key Players in Animal Longevity
Source: Aging Cell. 2025 Jun 28;24(9):e70156. doi: 10.1111/acel.70156 (PMC12419847; doi:10.1111/acel.70156)
Supplement: Supplementary file 2 — Figure S1. Covariation between maximum lifespan and weight values. A log–log plot illustrates the mathematical. Figure S2. Avian lifespan network. Network of protein–protein interactions across lifespan‐associated genes. [file ACEL-24-e70156-s001.pdf]

Supplementary Information

SI1. Supplementary Data

SD1. Multiple methods for describing longevity

So far, different metrics have been proposed in the literature to discriminate species according to their longevity. In this work, we adopted the LQ index, the distribution of MLS, and two extended longevity traits ELL and 3L (see Methods in the main text) to investigate whether any of these could help in identifying convergent molecular signals underlying long- and short-liveness in birds. By applying these methods to our dataset, we found that distinct methods labeled different species as long- or short-lived.

Notably, it is plausible that a lifespan category groups species sharing additional phenotypes besides longevity. For example, long-lived large-bodied (3L) species were almost all flightless (Electronic. Material 5). When investigating convergent molecular evolution, having simultaneously multiple convergent traits can be a confounding factor that may mislead the result interpretation. For this reason, and also because there was not a corresponding alternative method to identify short-lived species, we discarded both ELL and 3L extended longevity traits. Consequently, we tested the presence of convergent evolution only in long- and short-lived species labeled using the MLS distribution and the LQ index.

However, all the investigations of convergent molecular evolution associated with longevity phenotypes failed to identify significant results when using LQ-labeled species (see the github repository for TRACCER results obtained in test analyses and controls).

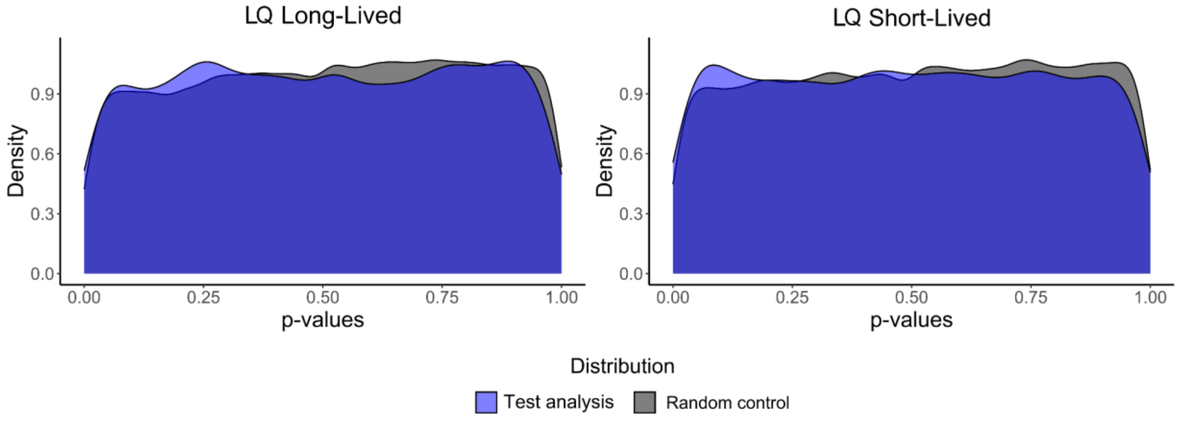

Supplementary Data Fig. 1 — Graphical representation of TRACCER results in LQ-labeled long- and short-lived species. The blue density curve represents the distribution of the p-values for the "test analyses" (i.e., conducted on long- or short-lived species labeled using the LQ index); the black curve represents the cumulative distribution of ten random controls. In both cases, genes with low p-values do not show any clear pattern of enrichment in the examined condition.

As shown in Supplementary Data Fig. 1, the convergent signal in trait-bearing species (i.e., the number of genes with a p-value lower than 0.05) is indeed not significantly different from what we could randomly expect. Therefore, different from what is obtained when using MLS to label long- and short-

lived species (see Results in the main text), long- and short-lived species identified with the LQ index do not show any signature of genomic convergence. A possible explanation for such a result is that no convergent evolution underlies species grouped based on their LQ. Therefore, in the study of longevity using convergent evolution methods, this index may not be appropriate because it could be, in contrast, more suitable for identifying species-specific aging traits and mechanisms.

Therefore, all the results reported in Supplementary Information and the main text, apart from this paragraph, concern only convergence identified using MLS as the longevity metric.

SD2. The problem of secondary convergent traits

When using MLS to define short- and long-lived species, a well-known confounding factor is the weight of species due to its positive correlation with maximum lifespan (Fig. 1 in the main text). Additionally, using MLS, we observed a bias in the taxonomic distribution of short-lived species since many of these belonged to the order Passeriformes. In such a scenario, Passeriformes-specific signals at the genomic level could have been erroneously interpreted as short-lived-specific signals.

To test if the convergence in our tested species might be due to other convergent traits than longevity, i.e., weight or biased phylogenetic distribution, we investigated the presence of a convergent evolution signal in two control datasets (see Methods in the main text and Electronic Materials 10-13); a "phylogenetic control", and a "weight control" per each longevity test analysis. We found significant signals of convergence (i.e., enrichment for low p-values with respect to distribution with randomly tagged species) in three out of four control datasets: the weight controls used for both long- and short-lived species (using heavy and light species based on the upper and the lower 25th percentiles of the weight distribution in our dataset, respectively) and the phylogenetic control used for short-lived species (mainly comprising birds belonging to the Passeriformes order) (Supplementary Data Fig. 2).

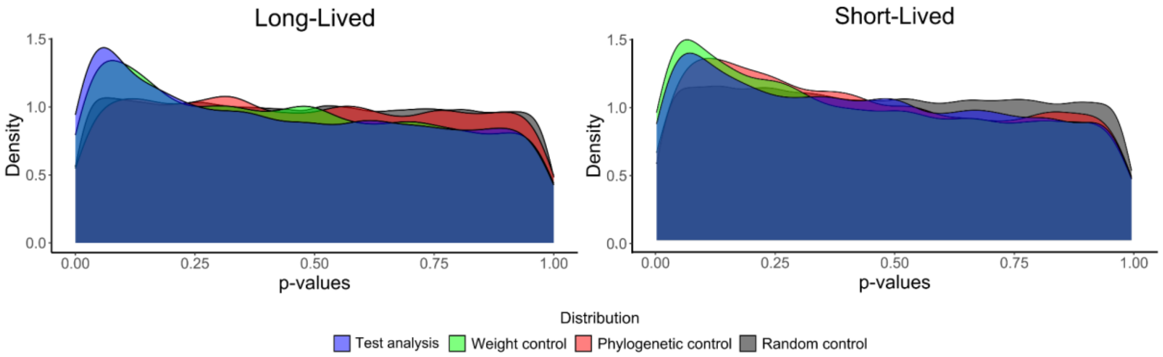

Supplementary Data Fig. 2. Graphical representation of TRACCER results comparing test analyses with corresponding control analyses. The blue density curve represents the distribution of the p-values for the "test analyses" (i.e., *conducted on long- or short-lived species). In contrast, the red curve corresponds to the phylogenetic control, the green curve to the weight control, and the black curve to the cumulative distribution of ten random controls. The left panel shows results for long-lived species, whereas the right panel displays results for short-lived species. The blue density curve indicates a significantly different distribution in long-lived species compared to the flat distributions of random and phylogenetic controls. However, it overlaps partially with the weight control distribution, which shows a peak in the leftmost part of the plot. This less left-skewness, though less pronounced than in the short-lived comparison, suggests a significant enrichment of convergently evolved genes. In contrast, while distinct from the random control distribution, the density curve for short-lived species is more left-skewed than the respective phylogenetic control but lower than its weight control. Still, its shape remains comparable to those of the controls.*

To investigate whether the convergent signal we observe in long- and short-lived species might be due to these secondary convergences, we assessed the overlap between convergence genes in test and control analyses.

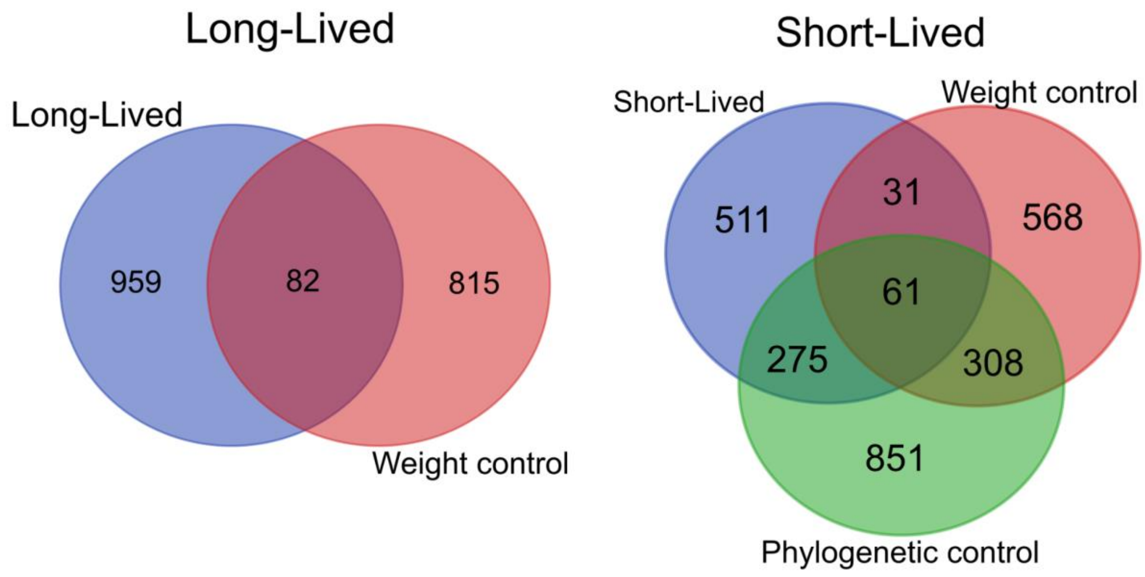

Supplementary Data Fig. 3. Venn diagrams showing the overlap between genes with convergent evolution in long-lived species and the corresponding weight control dataset (left) and between genes with convergent evolution in short-lived species and the corresponding weight and phylogenetic control datasets (right).

We found a minor proportion of genes showing convergent evolution in the tested longevity groups and their respective weight controls (6.1% and 15.3% for long- and short-lived species, respectively; Supplementary Data Fig. 3). Differently, we found that a considerable proportion of genes (39.6%) shows signatures of convergent evolution in both short-lived species and in its phylogenetic control. Such results show that particular attention is needed when analyzing convergent molecular evolution in convergent phenotypes to account for "concealed" shared features unrelated to the studied trait. To limit the possibility of analyzing genes showing convergent evolution associated with other traits besides longevity, we removed from subsequent analyses those genes showing convergent evolution in both test groups and any of the systematic controls. Overall, our findings highlight the importance of addressing biases such as dataset-specific features and taxonomic distribution, ensuring accurate interpretation of genomic signals in longevity studies.

SI2. Supplementary Figures

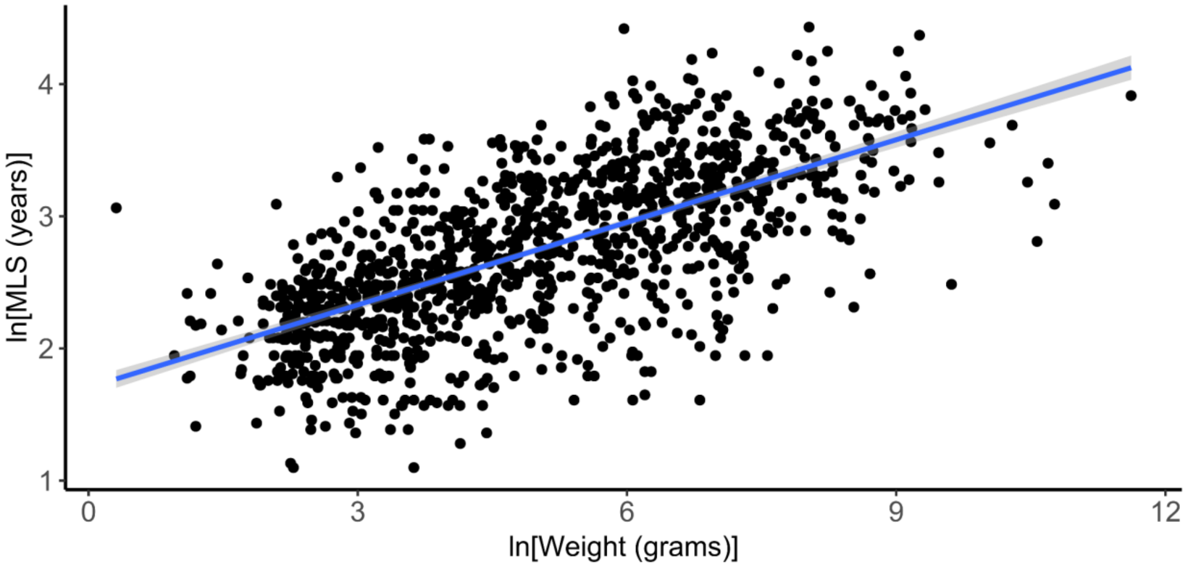

*Supplementary Fig. 1. Covariation between maximum lifespan and weight values. A log-log plot illustrates the mathematical covariation between maximum lifespan (MLS) (in years) and weight values (in grams) in birds. The blue line represents the fitted linear regression model, described by the equation MLS = 5.5027 * weight$^{0.2082}$, with R^2 = 0.4456 (Supplementary Table 1).*

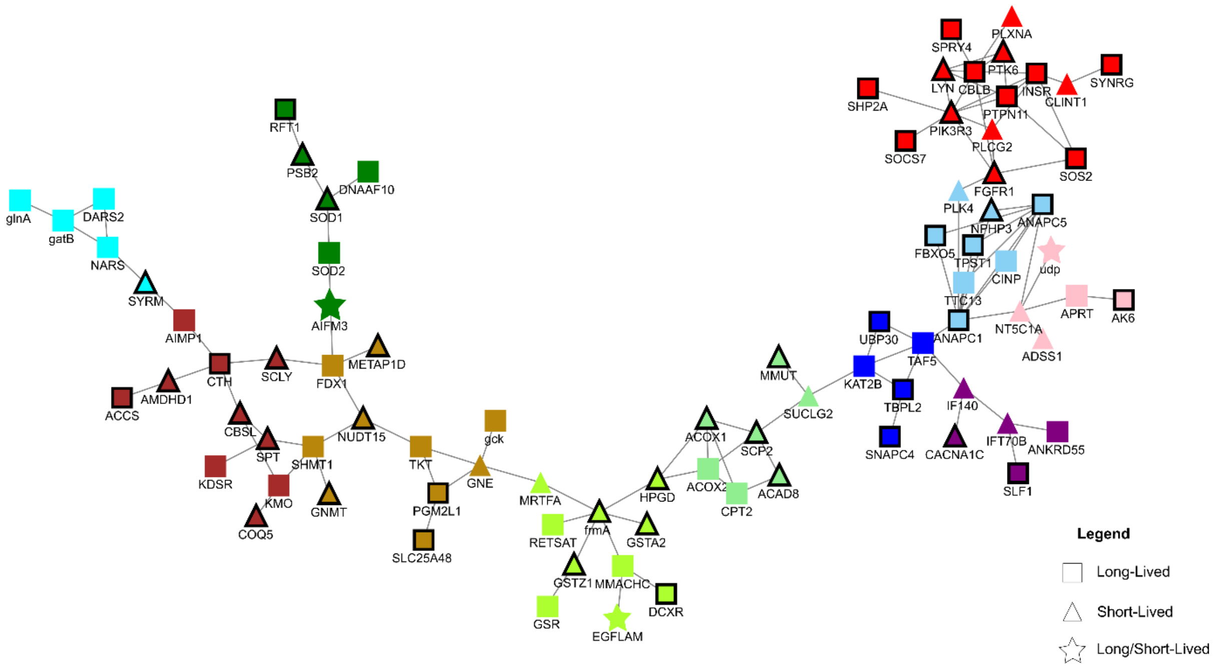

Supplementary Fig. 2. Avian lifespan network. Network of protein-protein interactions across lifespan-associated genes in birds. Nodes represent proteins, edges represent interactions. Colours correspond to the eleven modules inferred using the k-means clustering algorithm in STRING. Square-shaped nodes represent proteins showing convergent evolution in long-lived species, triangle-shaped nodes represent proteins showing convergent evolution in short-lived species, while star-shaped nodes represent proteins with convergent evolution in both long- and short-lived analysis, but with opposite evolutionary forces. Bordered nodes identify constrained genes, while nodes without a border are accelerated genes. .

SI3. Supplementary Tables

**Intercept**	**Inclination**	**R^2**
5.5027	0.2082	0.4456

Supp. Table 1. Parameters of the linear regression model describing the mathematical relationship between maximum lifespan (MLS) and weight values (see Supp. Fig. 1).

**HAGR databases**	**Observed**	**Expected**	**$\chi 2$**	**p-value**
**lifespan network vs. other networks**	32	21	14.3667143	0.000150438 *
**lifespan network vs. all lifespan genes**	32	14	35.30551893	2.81832E-09 *
**lifespan network vs. all orthogroups**	32	15	23.35177947	1.34919E-06 *
**hub genes vs. other genes in lifespan network**	6	4	2.207369729	0.137353

Supp. Table 2. Results of the $\chi 2$ test for the overrepresentation of genes included in the HAGR databases among genes of interest. We tested the overrepresentation of genes included in the HAGR databases present in our lifespan network compared to those in all other networks, in all lifespan genes, and in all orthogroups analyzed in this work. Additionally, we tested the overrepresentation of genes included in the HAGR databases in hub genes compared to non-hub genes within the lifespan network. Columns report the number of observed and expected genes from the HAGR databases, the $\chi 2$ statistic, and the p-value. Significant p-values are marked by "".*

**H-GenAge database**	**Observed**	**Expected**	**$\chi 2$**	**p-value**
**lifespan network vs. other network**	10	5	11.36914035	0.000746744 *
**lifespan network vs. all lifespan genes**	10	2	39.82039133	2.78423E-10 *
**lifespan network vs. all orthogroups**	10	2	27.02309524	2.01039E-07 *
**hub genes vs. other genes in lifespan network**	4	1	8.383171327	0.003787 *

Supp. Table 3. Results of the $\chi 2$ test for the overrepresentation of genes included in the Human GenAge (H-GenAge) database among genes of interest. We tested the overrepresentation of genes included in the H-GenAge database in our lifespan network compared to those present in all other networks, in all lifespan genes, and in all orthogroups analyzed in this work. Additionally, we tested the overrepresentation of genes included in the H-GenAge database in hub genes compared to non-hub genes within the lifespan network. Columns report the number of observed and expected genes from the H-GenAge database, the $\chi 2$ statistic, and the p-value. Significant p-values are marked by "".*
